# Supplementary material for: Remodelling of the gut microbiota by hyperactive NLRP3 induces regulatory T cells to maintain homeostasis
Source: Nat Commun. 2017 Dec 1;8:1896. doi: 10.1038/s41467-017-01917-2 (PMC5711854; doi:10.1038/s41467-017-01917-2)
Supplement: Supplementary file 1 — Supplementary Information [file 41467_2017_1917_MOESM1_ESM.pdf]

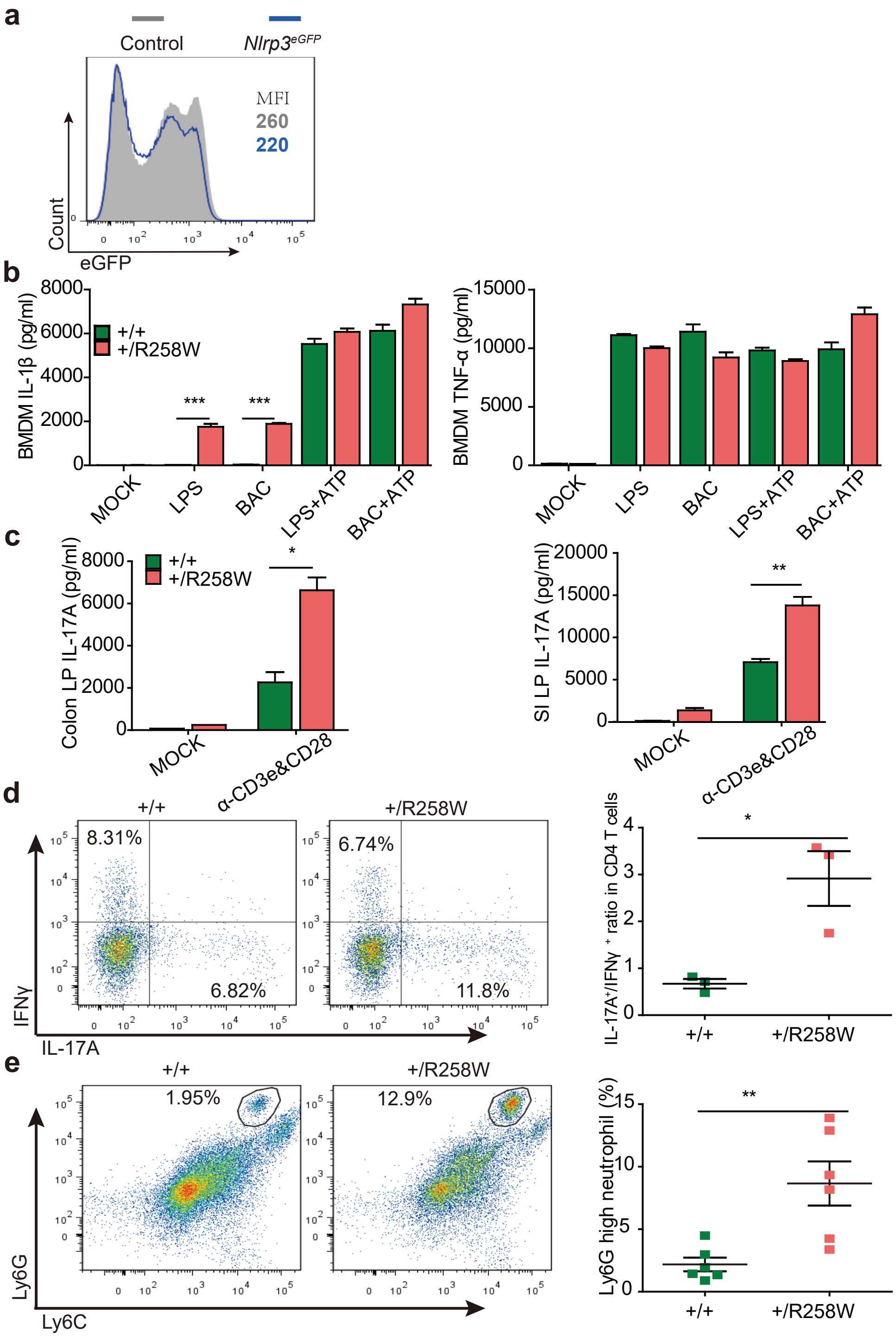

a
Control
Nlrp3eGFP
MFI
260
220
Count
eGFP
b
BMDM IL-1β (pg/ml)
+/+
+/R258W
***
***
MOCK
LPS
BAC
LPS+ATP
BAC+ATP
BMDM TNF-α (pg/ml)
c
Colon LP IL-17A (pg/ml)
+/+
+/R258W
*
MOCK
α-CD3e&CD28
SI LP IL-17A (pg/ml)
**
d
+/+
+/R258W
8.31%
6.82%
6.74%
11.8%
IFNγ
IL-17A
IL-17A+/IFNγ+ ratio in CD4 T cells
*
e
1.95%
12.9%
Ly6G
Ly6C
Ly6G high neutrophil (%)
**

Supplementary Figure 1. *Nlrp3*$^{\mathbf{R258W}}$ mice contain a hyperactive inflammasome in the intestine, related to Figure 1.

(**a**) The expression of NLRP3 reflected by eGFP signal in colonic epithelial cells isolated from control (grey) and *Nlrp3*^{eGFP} reporter mice (blue) were analyzed with flowcytometry. MFI represents geomean fluorescence intensity of eGFP.

(**b**) WT and *Nlrp3*^{R258W} BMDMs were stimulated with heat inactivated faecal bacteria (BAC) at multiplicity of infection (MOI)=20:1 or LPS (100 ng/ml) for 5.5h with or without ATP (5mM) pulse for 30min, IL-1β and TNF-α from cell culture supernatant were determined via ELISA.

(**c**) Lamina propria (LP) cells isolated from the colon (left panel) or small intestine (right panel) of indicated mice were reactivated with anti-CD3e (1ug/ml) + anti-CD28 (0.25ug/ml) for 48h, IL-17A from culture supernatant was determined via ELISA.

(**d-e**) Colonic lamina propria cells were isolated from WT and *Nlrp3*^{R258W} mice, stimulated with PMA (50ng/ml) plus ionomycin (500ng/ml) in the presence of GolgiStop (0.67ul/ml) for 4h, followed with staining for CD4, IL-17A and IFN-γ, then the ratio of IL-17A^{+}/IFN-γ^{+} in CD4^{+} T cells were depicted in (**d**); or directly stained for CD11b, Ly6G and Ly6C, the percentage of Ly6G^{high} in CD11b^{+} cells were displayed in (**e**). (**b**-**e**) Data are shown as the means ± SEM. * *P*< 0.05, ** *P*< 0.01, *** *P*< 0.001 (Two tailed student's t-test).

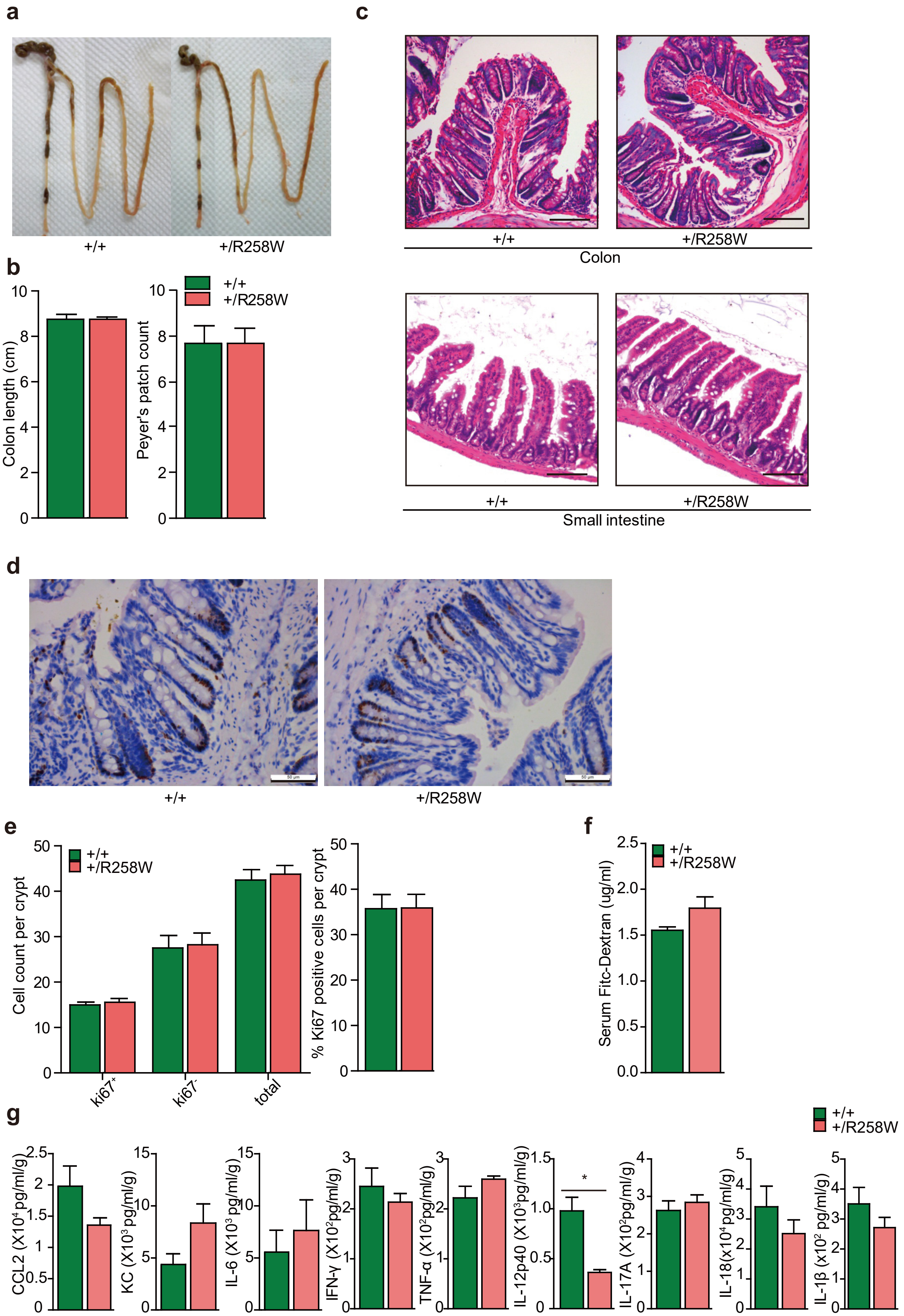
a
+/+
+/R258W
b
Colon length (cm)
Peyer's patch count
+/+
+/R258W
c
+/+
+/R258W
Colon
+/+
+/R258W
Small intestine
d
+/+
+/R258W
e
Cell count per crypt
+/+
+/R258W
ki67+
ki67-
total
% Ki67 positive cells per crypt
f
Serum Fitc-Dextran (ug/ml)
+/+
+/R258W
g
+/+
+/R258W
CCL2 (X10⁴pg/ml/g)
KC (X10³pg/ml/g)
IL-6 (X10³pg/ml/g)
IFN-γ (X10²pg/ml/g)
TNF-α (X10²pg/ml/g)
IL-12p40 (X10³pg/ml/g)
*
IL-17A (X10²pg/ml/g)
IL-18(x10⁴pg/ml/g)
IL-1β (x10² pg/ml/g)

Supplementary Figure 2. ***Nlrp3*$^{\mathbf{R258W}}$ mice maintain intestinal homeostasis.**

(**a**) Photographic comparison of intestines from WT (+/+) and *Nlrp3*^{R258W} (+/R258W) mice under resting status.

(**b**) Colon length (left panel) and Peyer's patch counts (right panel) from WT and *Nlrp3*^{R258W} mice.

(**c**) Hematoxylin and eosin (H&E) examination of colon (upper panel) and small intestine (lower panel) mucosa structure from WT and *Nlrp3*^{R258W} mice. Scale bar, 100um.

(**d-e**) Paraffin sections of colon from WT and *Nlrp3*^{R258W} mice were stained with antibody against Ki67 (**d**), scale bar, 50um; and the positive cell numbers and percentage per crypt were assessed quantitatively (**e**).

(**f**) Untreated WT (n=4) and *Nlrp3*^{R258W} (n=4) mice were gavaged with FITC-Dextran and leakage of FITC-Dextran into the serum was measured 4h later.

(**g**) Indicated inflammatory mediators were determined via ELISA for colon tissue culture supernatants from untreated WT and *Nlrp3*^{R258W} mice. (**b, e-g**) Data are shown as means ± SEM. * $P < 0.05$ (Two tailed student's t-test).

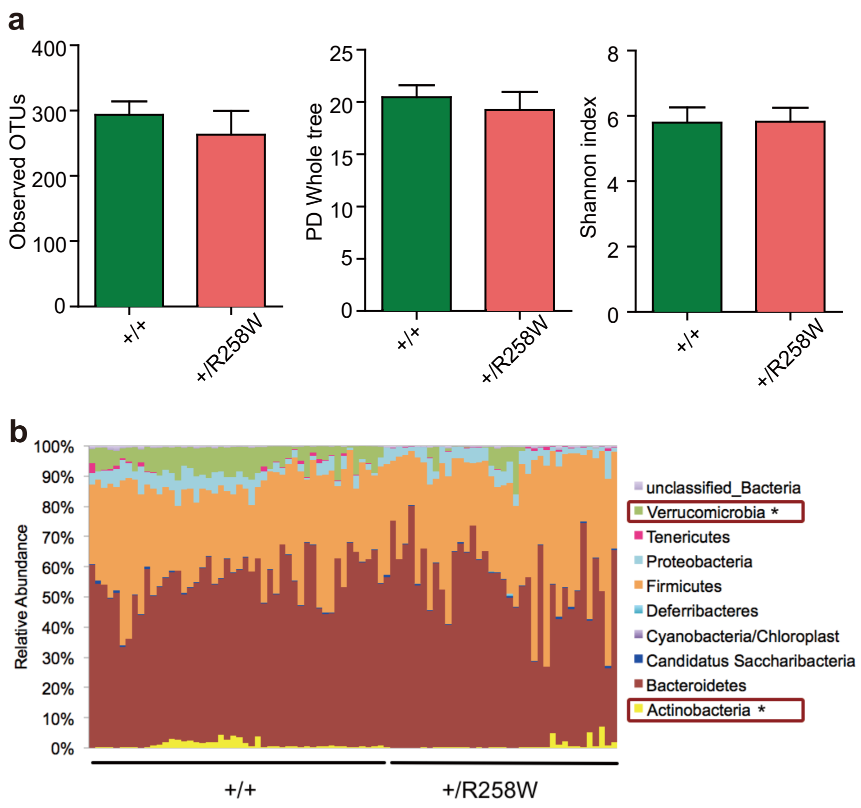
a
400
300
200
100
0
Observed OTUs
+/+
+/R258W
25
20
15
10
5
0
PD Whole tree
+/+
+/R258W
8
6
4
2
0
Shannon index
+/+
+/R258W
b
100%
90%
80%
70%
60%
50%
40%
30%
20%
10%
0%
Relative Abundance
unclassified_Bacteria
Verrucomicrobia *
Tenericutes
Proteobacteria
Firmicutes
Deferribacteres
Cyanobacteria/Chloroplast
Candidatus Saccharibacteria
Bacteroidetes
Actinobacteria *
+/+
+/R258W

Supplementary Figure 3. *Nlrp3*^{R258W} mice carry a unique gut microbiota, related to Figure 3.

(**a-b**) WT and *Nlrp3*^{R258W} mice fecal bacteria 16S rRNA V3-V4 region was sequenced with Miseq technology (Illumina), the total operative taxonomic units (OTUs), PD whole tree and Shannon index indicating α-diversity were compared between these mice; data are shown as means ± SEM (**a**). The composition of microbiota from WT and *Nlrp3*^{R258W} mice at phylum level was also compared, *$P<0.05$, Mann-Whitney test (**b**).

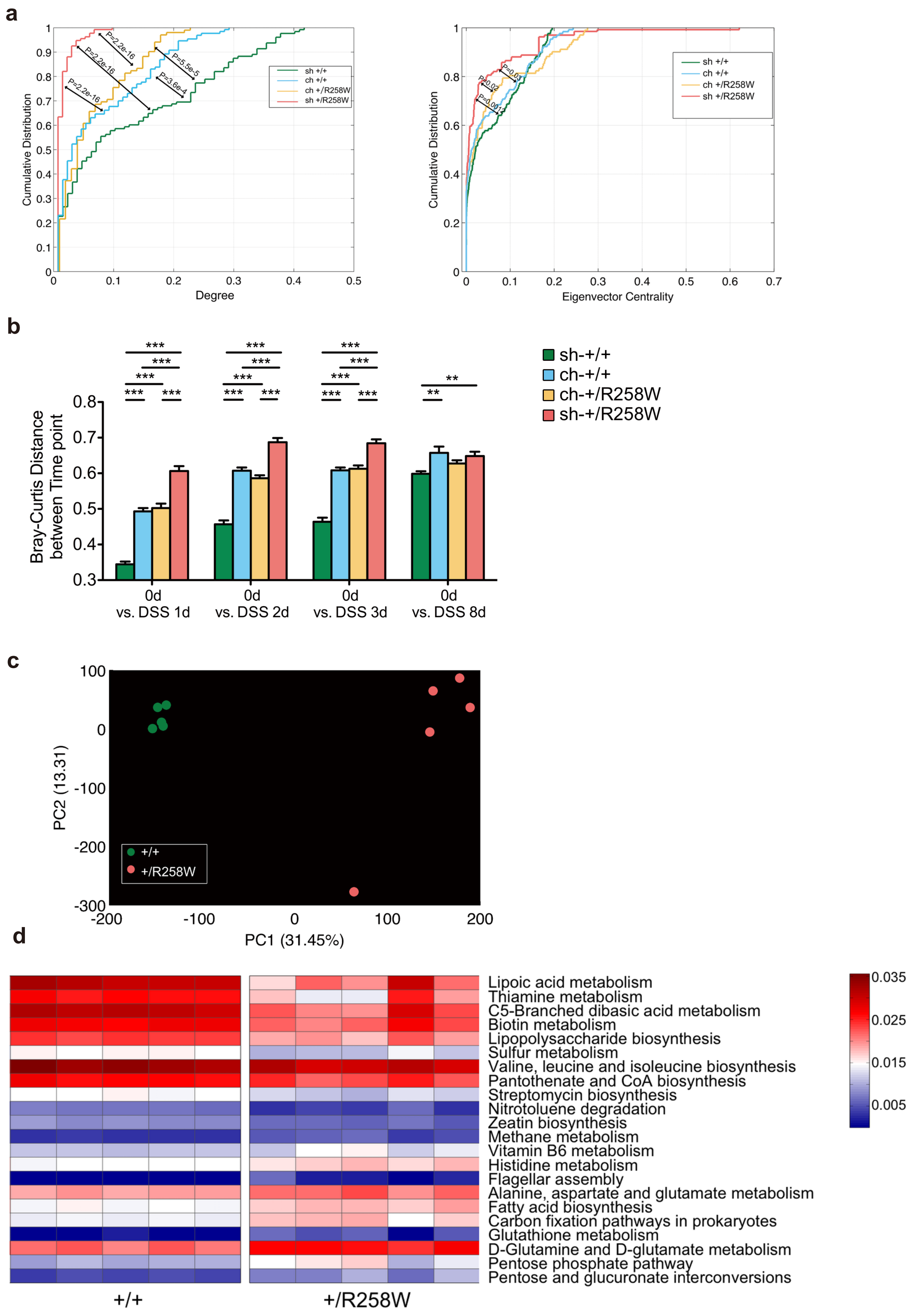
a
Cumulative Distribution
Degree
sh +/+
ch +/+
ch +/R258W
sh +/R258W
P=2.2e-16
P=2.2e-16
P=2.2e-16
P=5.5e-5
P=3.6e-4
Eigenvector Centrality
P=0.03
P=0.02
P=0.0012
b
Bray-Curtis Distance between Time point
sh-+/+
ch-+/+
ch-+/R258W
sh-+/R258W
0d vs. DSS 1d
0d vs. DSS 2d
0d vs. DSS 3d
0d vs. DSS 8d
c
PC2 (13.31)
PC1 (31.45%)
+/+
+/R258W
d
Lipoic acid metabolism
Thiamine metabolism
C5-Branched dibasic acid metabolism
Biotin metabolism
Lipopolysaccharide biosynthesis
Sulfur metabolism
Valine, leucine and isoleucine biosynthesis
Pantothenate and CoA biosynthesis
Streptomycin biosynthesis
Nitrotoluene degradation
Zeatin biosynthesis
Methane metabolism
Vitamin B6 metabolism
Histidine metabolism
Flagellar assembly
Alanine, aspartate and glutamate metabolism
Fatty acid biosynthesis
Carbon fixation pathways in prokaryotes
Glutathione metabolism
D-Glutamine and D-glutamate metabolism
Pentose phosphate pathway
Pentose and glucuronate interconversions
+/+
+/R258W

Supplementary Figure 4. The microbiota from WT and *Nlrp3*^{R258W} mice differs in the microbial ecology and functionality, related to Figure 3 and 4.

(**a**) The degree and eigenvector centralities were calculated for the network of OTUs from sh-+/+, ch-+/+, ch-+/R258W and sh-+/R258W mice during DSS treatment (shown in Figures 3f, 4f, respectively). P-Values to test for differences in cumulative distributions were obtained by one-sided Kolmogorov-Smirnov tests.

(**b**) The Bray-Curtis distance of the gut microbiota before DSS challenge (0d) to the gut microbiota after DSS challenge (1d, 2d, 3d and 8d) was calculated. The data are shown as means ± SEM. ** $P< 0.01$, *** $P< 0.001$ (One-way ANOVA with Tukey's post-hoc test).

(**c**) The PCA score plot of the KEGG orthology (KO) groups recognized with HUMAnN showing a significant shift of the KO profiles between WT and *Nlrp3*^{R258W} microbiota (log-transformed).

(**d**) The heat map shows the abundance of the key pathways in Fig. 3g in each mouse.

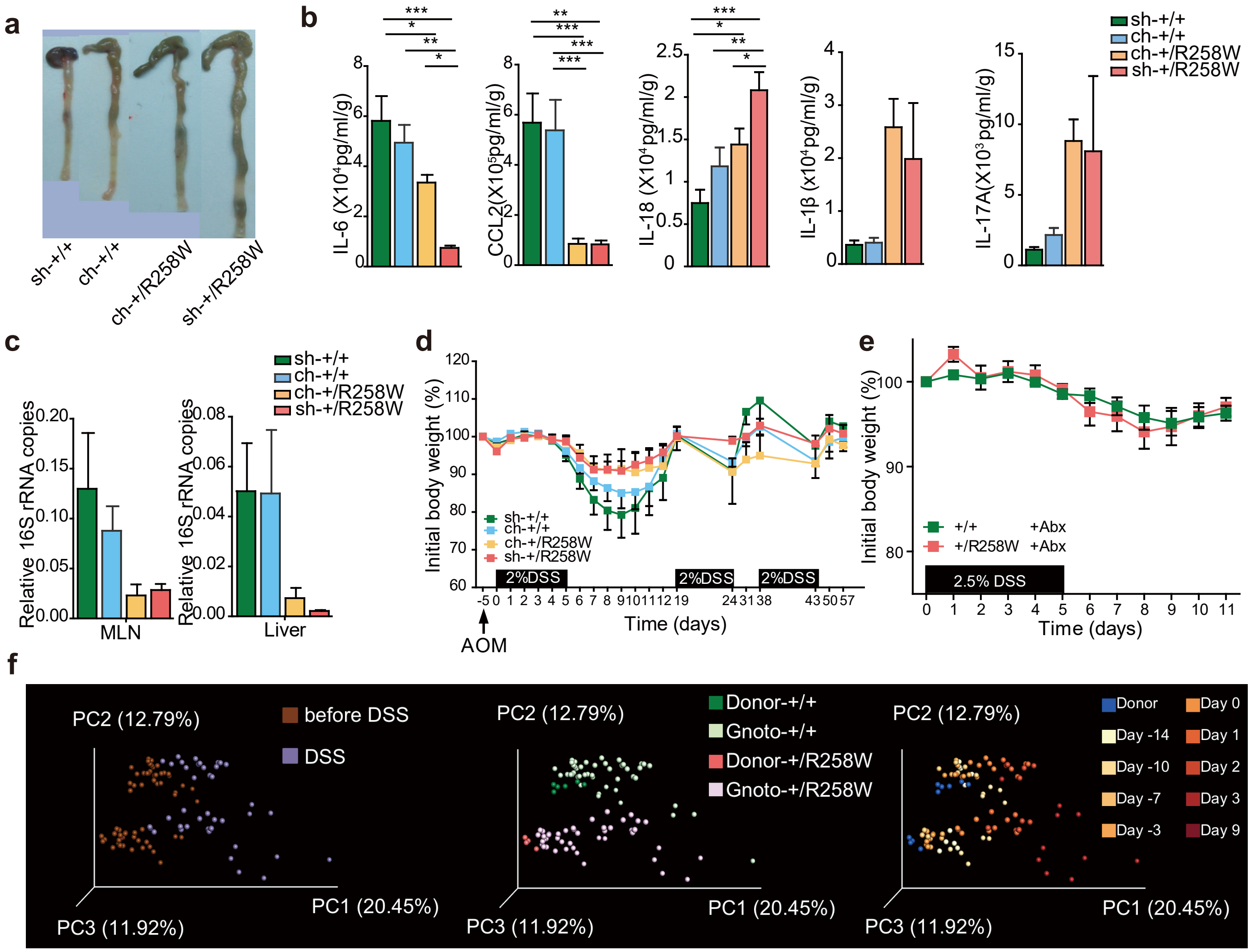

a
sh-+/+
ch-+/+
ch-+/R258W
sh-+/R258W
b
IL-6 (X10⁴pg/ml/g)
CCL2(X10⁵pg/ml/g)
IL-18 (X10⁴pg/ml/g)
IL-1β (x10⁴pg/ml/g)
IL-17A(X10³pg/ml/g)
c
Relative 16S rRNA copies
MLN
Liver
d
Initial body weight (%)
2%DSS
Time (days)
AOM
e
+/+ +Abx
+/R258W +Abx
2.5% DSS
Time (days)
f
PC1 (20.45%)
PC2 (12.79%)
PC3 (11.92%)
before DSS
DSS
Donor-+/+
Gnoto-+/+
Donor-+/R258W
Gnoto-+/R258W
Donor
Day -14
Day -10
Day -7
Day -3
Day 0
Day 1
Day 2
Day 3
Day 9

Supplementary Figure 5. Gut microbiota affects DSS colitis and colorectal cancer development, related to Figure 5.

(**a-b**) Colitis was induced in mice as indicated in Figures 5a-5e, the representative colons (**a**) and inflammatory mediators determined by ELISA in colon tissue culture supernatants (**b**) were shown. (**c**) Bacteria dissemination to mesenteric lymphnode (MLN) and liver was determined via Q-PCR with 16S rRNA universal primer pair from indicated mice. (**d**) Weight curve of mice from colon cancer experiment in Figures 5f-5g were displayed. (**e**) WT and *Nlrp3*^{R258W} mice were treated with antibiotics cocktail (Abx) to remove intestinal bacteria before being subjected to DSS colitis experiment, body weight during DSS colitis induction was assessed. (**f**) The same mice in Figures 5h-5k, the gut microbiota from donor and recipient mice were plotted in PCoA graph based on Bray-Curtis Distance. Data are shown as mean ± SEM (**b-e**), * $P<0.05$, ** $P<0.01$, *** $P<0.001$ (One-way ANOVA with Tukey's post-hoc test).

Supplementary Figure 6.

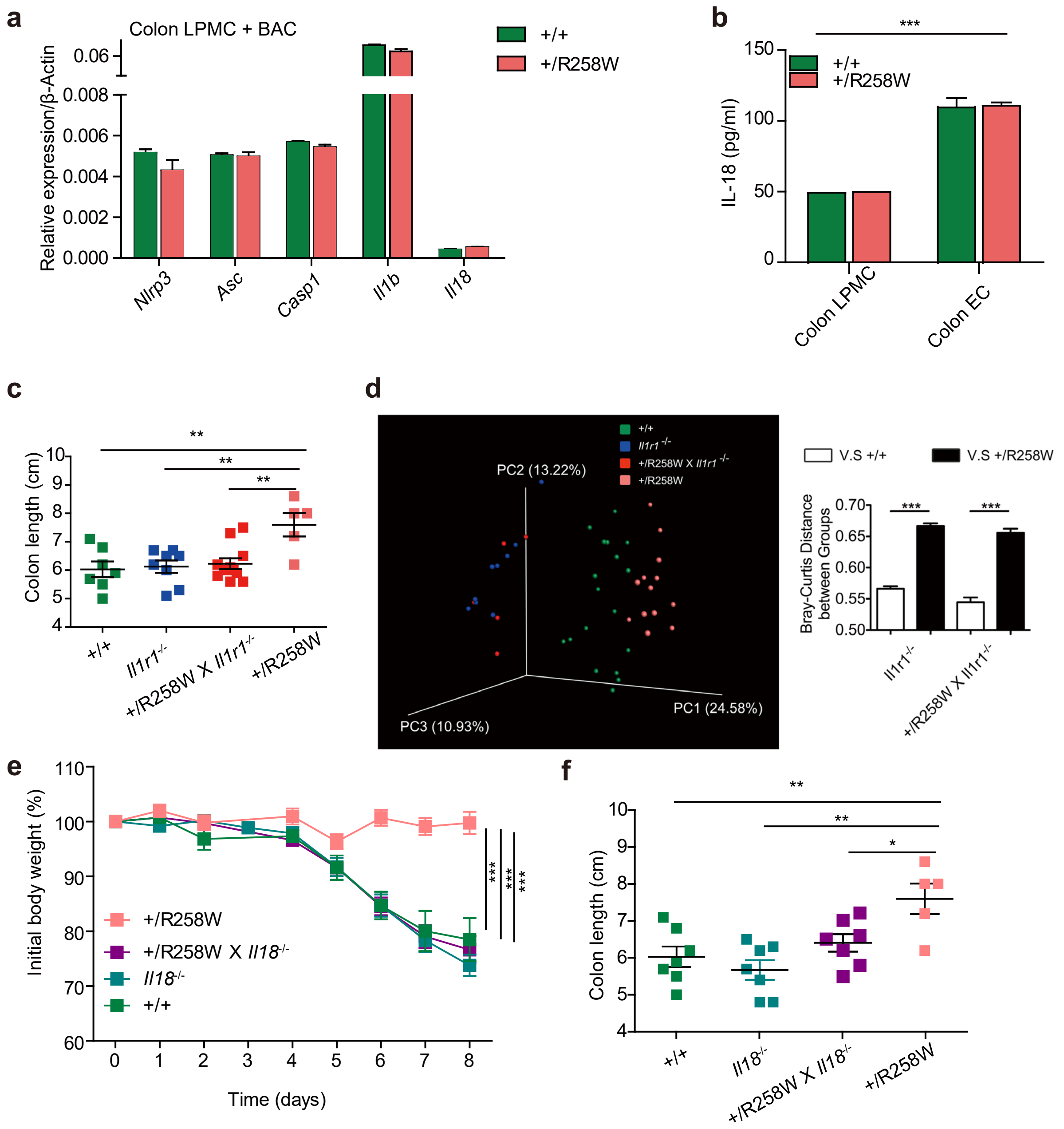

Supplementary Figure 6. *Nlrp3^{R258W}* mutation shapes microbiota via IL-1β, related to Figure 6.

(**a**) Colonic LPMCs isolated from WT and *Nlrp3^{R258W}* mice were stimulated with heat-inactivated mouse faecal bacteria (BAC) (MOI=20:1) for 12h and assayed for the expression of indicated genes via Q-PCR.

(**b**) Isolated colon LPMC and EC cells as in Figures 6a-6b from both WT and *Nlrp3^{R258W}* mice were cultured without stimulation for 24h, supernatant IL-18 was determined via ELISA. ***P<0.001 (Two tailed student's t-test).

(**c**) As in Figure 6e, the colon length was measured on day 8 upon sacrifice of indicated experimental mice. **P<0.01 (One-way ANOVA with Tukey's post-hoc test).

(**d**) The Bray-Curtis distance of microbiota from *Il1r1$^{-/-}$* and *Nlrp3^{R258W}* x *Il1r1$^{-/-}$* mice to that from WT or *Nlrp3^{R258W}* mice were calculated. ***P<0.001 (Two tailed student's t-test).

(**e**) Changes of body weight in WT (n=8), *Il18$^{-/-}$* (n=10), *Nlrp3*^{R258W} x *IL18$^{-/-}$* (n=7) and *Nlrp3*^{R258W} (n=5) mice during DSS-induced colitis. ***P<0.001 (One-way ANOVA with Tukey's post-hoc test).

(**f**) The colon length of mice in (**e**) was monitored. *P<0.05, **P<0.01 (One-way ANOVA with Tukey's post-hoc test).

Supplementary Figure 7.

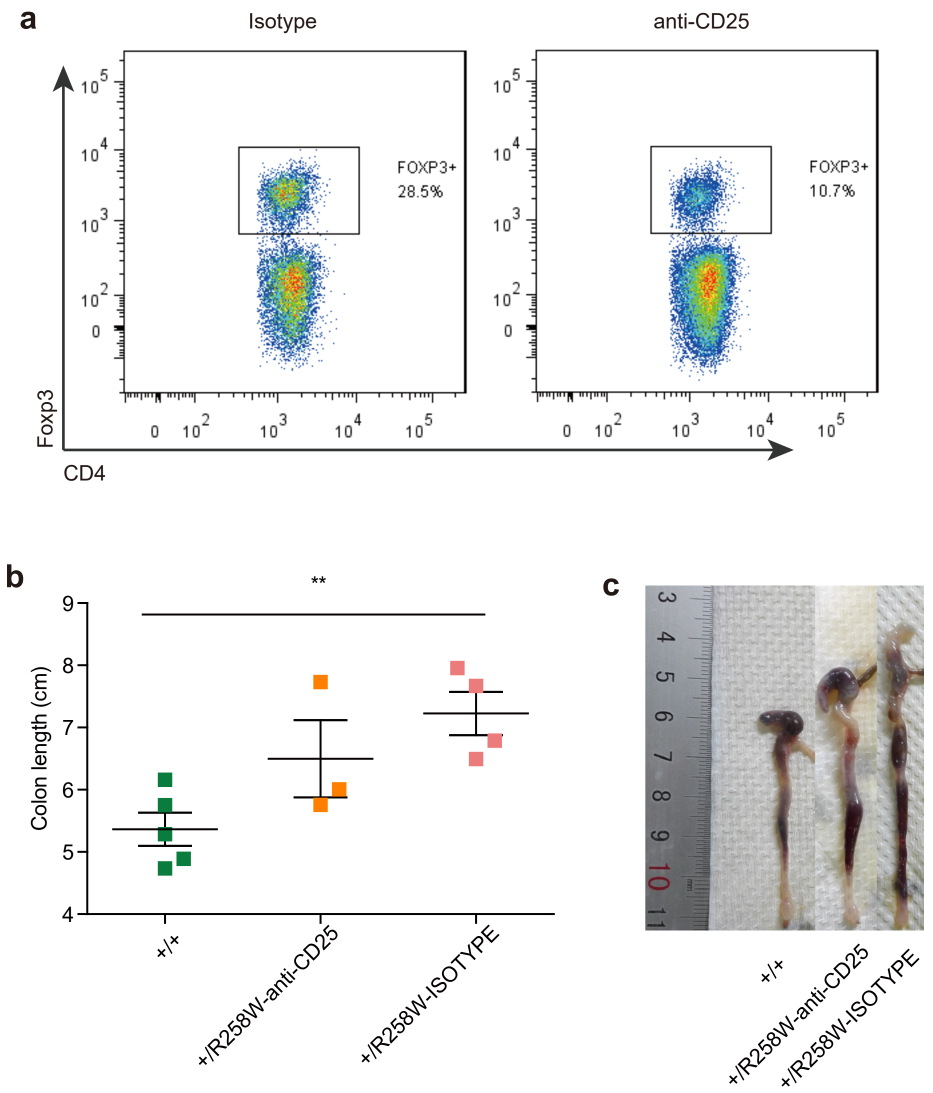

Supplementary Figure 7. Depletion of Tregs in *Nlrp3*^{R258W} mice abolishes protection against DSS colitis, related to Figure 7.

(**a-c**) WT (n=5) untreated mice, *Nlrp3*^{R258W} (n=4) mice i.p. injected with isotype antibody, and *Nlrp3*^{R258W} (n=6) mice i.p. injected with anti-CD25 antibody on day-4 and day-2 with 500ug/mouse/injection were induced for colitis with 4% DSS from day0. The depletion efficiency for Tregs was monitored by FACS, showing representative data (**a**). The final colon length was summarized in (**b**) and the representative colons were shown in (**c**). The data are shown as the means ± SEM, *$P<0.05$ (One-way ANOVA with Dunnett's post-hoc test).

Supplementary Figure 8.

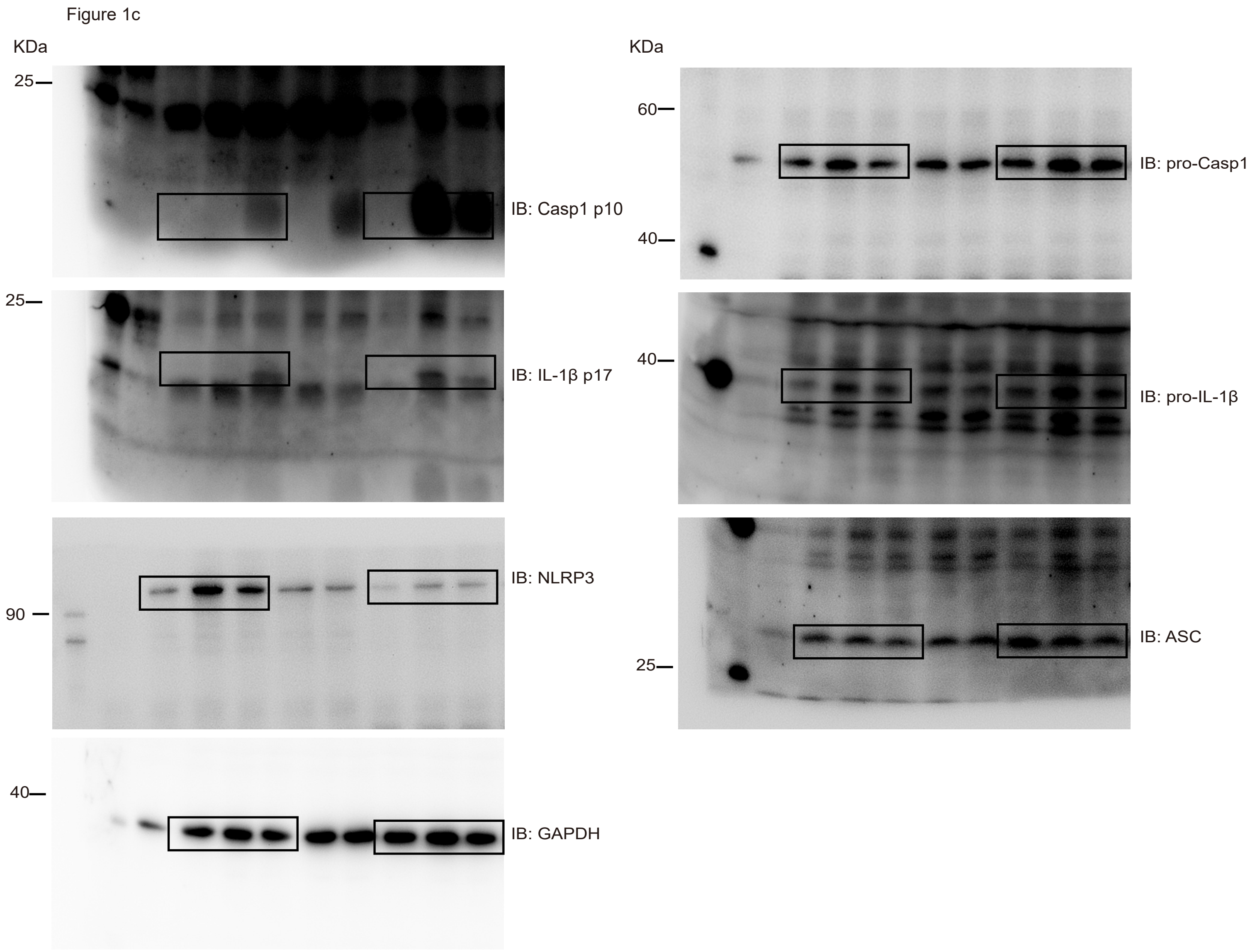

Supplementary Figure 8. Full gel images for the data shown in Figure 1c.

Areas in rectangles marked on the gel images are the selected areas showed in Figure 1c, displaying cleaved caspase-1 p10 subunit, pro-caspase-1, IL-1β-p17 fragment, pro-IL-1β, NLRP3, ASC and GAPDH.

Supplemetary Table 1. 50 key OTUs that were signifcantly different between WT and *Nlrp3*^{R258W} mice gut microbiota, related to Fig. 3c.

Serial NO	OTUs	Phylum	Class	Order	Family	Genus
1	OTU78	Bacteroidetes	Bacteroidia	Bacteroidales	Porphyromonadaceae	Barnesiella
2	OTU66	Proteobacteria	Epsilonproteobacteria	Campylobacterales	Helicobacteraceae	Helicobacter
3	OTU18	Bacteroidetes	Bacteroidia	Bacteroidales	Porphyromonadaceae	unclassified_Porphyromonadaceae
4	OTU29	Bacteroidetes	Bacteroidia	Bacteroidales	Porphyromonadaceae	unclassified_Porphyromonadaceae
5	OTU23	Bacteroidetes	Bacteroidia	Bacteroidales	Porphyromonadaceae	unclassified_Porphyromonadaceae
6	OTU175	Bacteroidetes	Bacteroidia	Bacteroidales	Porphyromonadaceae	unclassified_Porphyromonadaceae
7	OTU52	Bacteroidetes	Bacteroidia	Bacteroidales	unclassified_Bacteroidales	
8	OTU77	Bacteroidetes	Bacteroidia	Bacteroidales	Porphyromonadaceae	unclassified_Porphyromonadaceae
9	OTU7	Bacteroidetes	uclassified_Bacteroidetes			
10	OTU30	Bacteroidetes	Bacteroidia	Bacteroidales	Bacteroidaceae	Bacteroides
11	OTU15	Bacteroidetes	Bacteroidia	Bacteroidales	Porphyromonadaceae	unclassified_Porphyromonadaceae
12	OTU136	Bacteroidetes	Bacteroidia	Bacteroidales	Porphyromonadaceae	unclassified_Porphyromonadaceae
13	OTU38	Bacteroidetes	Bacteroidia	Bacteroidales	Prevotellaceae	Alloprevotella
14	OTU112	Bacteroidetes	Bacteroidia	Bacteroidales	Porphyromonadaceae	unclassified_Porphyromonadaceae
15	OTU40	Bacteroidetes	Bacteroidia	Bacteroidales	Porphyromonadaceae	unclassified_Porphyromonadaceae
16	OTU41	Bacteroidetes	Bacteroidia	Bacteroidales	Porphyromonadaceae	unclassified_Porphyromonadaceae
17	OTU9	Firmicutes	Bacilli	Lactobacillales	Lactobacillaceae	Lactobacillus
18	OTU164	Firmicutes	Clostridia	Clostridiales	Lachnospiraceae	unclassified_Lachnospiraceae
19	OTU87	Firmicutes	Clostridia	Clostridiales	Ruminococcaceae	Butyricicoccus
20	OTU537	Firmicutes	Clostridia	Clostridiales	Lachnospiraceae	unclassified_Lachnospiraceae
21	OTU140	Firmicutes	Clostridia	Clostridiales	Lachnospiraceae	unclassified_Lachnospiraceae
22	OTU118	Firmicutes	Clostridia	Clostridiales	Lachnospiraceae	unclassified_Lachnospiraceae
23	OTU304	Firmicutes	Clostridia	Clostridiales	Lachnospiraceae	unclassified_Lachnospiraceae
24	OTU64	Firmicutes	Clostridia	Clostridiales	Lachnospiraceae	Clostridium XlVa
25	OTU575	Firmicutes	Clostridia	Clostridiales	Lachnospiraceae	Clostridium XlVa
26	OTU163	Firmicutes	Clostridia	Clostridiales	Lachnospiraceae	unclassified_Lachnospiraceae
27	OTU178	Firmicutes	Clostridia	Clostridiales	Lachnospiraceae	unclassified_Lachnospiraceae
28	OTU365	Firmicutes	Clostridia	Clostridiales	Lachnospiraceae	unclassified_Lachnospiraceae
29	OTU668	Firmicutes	Clostridia	Clostridiales	Lachnospiraceae	unclassified_Lachnospiraceae
30	OTU61	Bacteroidetes	Bacteroidia	Bacteroidales	Prevotellaceae	unclassified_Prevotellaceae
31	OTU21	Bacteroidetes	Bacteroidia	Bacteroidales	Porphyromonadaceae	Odoribacter
32	OTU53	Firmicutes	Clostridia	Clostridiales	unclassified_Clostridiales	
33	OTU4	Firmicutes	Erysipelotrichia	Erysipelotrichales	Erysipelotrichaceae	unclassified_Erysipelotrichaceae
34	OTU142	Firmicutes	Clostridia	Clostridiales	Lachnospiraceae	unclassified_Lachnospiraceae
35	OTU20	Bacteroidetes	Bacteroidia	Bacteroidales	Prevotellaceae	Prevotella
36	OTU28	Bacteroidetes	Bacteroidia	Bacteroidales	Prevotellaceae	Prevotella
37	OTU16	Firmicutes	Clostridia	Clostridiales	Lachnospiraceae	unclassified_Lachnospiraceae
38	OTU63	Firmicutes	Clostridia	Clostridiales	unclassified_Clostridiales	
39	OTU34	Actinobacteria	Actinobacteria	Coriobacteriales	Coriobacteriaceae	Olsenella
40	OTU13	Bacteroidetes	Bacteroidia	Bacteroidales	Porphyromonadaceae	unclassified_Porphyromonadaceae
41	OTU88	Bacteroidetes	Bacteroidia	Bacteroidales	Porphyromonadaceae	unclassified_Porphyromonadaceae
42	OTU105	Proteobacteria	Deltaproteobacteria	Desulfovibrionales	Desulfovibrionaceae	unclassified_Desulfovibrionaceae
43	OTU1	Verrucomicrobia	Verrucomicrobiae	Verrucomicrobiales	Verrucomicrobiaceae	Akkermansia
44	OTU48	Firmicutes	Bacilli	Lactobacillales	Lactobacillaceae	Lactobacillus
45	OTU45	Bacteroidetes	Bacteroidia	Bacteroidales	Porphyromonadaceae	unclassified_Porphyromonadaceae
46	OTU99	Bacteroidetes	Bacteroidia	Bacteroidales	Porphyromonadaceae	unclassified_Porphyromonadaceae
47	OTU33	Bacteroidetes	Bacteroidia	Bacteroidales	Porphyromonadaceae	unclassified_Porphyromonadaceae
48	OTU2	Bacteroidetes	Bacteroidia	Bacteroidales	Porphyromonadaceae	unclassified_Porphyromonadaceae
49	OTU431	Bacteroidetes	Bacteroidia	Bacteroidales	Porphyromonadaceae	Barnesiella
50	OTU6	Bacteroidetes	Bacteroidia	Bacteroidales	Porphyromonadaceae	unclassified_Porphyromonadaceae

Supplementary Table 2. Network parameters, related to Fig. 3f and 4f.

	sh-+/+	ch-+/+	ch-+/R258W	sh-+/R258W
**Number of samples**	38	40	42	42
**Number of OTUs**[a]	299	301	288	268
**Number of nodes (|R|>0.7)**	128	130	102	134
**Isolated nodes (%)**[b]	22.7	23.1	21.6	63.4
**Clustering coefficient**	0.522	0.484	0.459	0
**Connected components**	7	12	7	11
**Network centralization**	0.295	0.224	0.167	0.085
**Characteristic path length**	2.995	3.146	3.544	7.151
**Average number of neighbors**	16.172	9.6	6.471	1.925
**Network density**	0.127	0.074	0.064	0.014

[a]OTUs shared by more than 40% samples in each group.

[b]The number of edge for one node =1

Supplementary Table 3. Primer List used in this study.

primer name	sequence (5'-3')
mNLRP3 FWD	TCTATGGTATGCCAGGAGGA
mNLRP3 REV	GCAGTTCACCAGTCTTTCTT
mASC FWD	CAGAGTACAGCCAGAACAGGACAC
mASC REV	GTGGTCTCTGCACGAACTGCCTG
mIL-18 FWD	GACTCTTGCGTCAACTTCAAGG
mIL-18 REV	CAGGCTGTCTTTTGTCAACGA
mCASP1 FWD	ACATTACTGCTATGGACAAGGC
mCASP1 REV	GCTGATGGAGCTGATTGAAG
mIL-1β FWD	GAAATGCCACCTTTTGACAG
mIL-1β REV	CTGGATGCTCTCATCAGGAC
mVillin-1 FWD	ACGCAGGTTGAGGTGCAGA
mVillin-1 REV	GGTTGGGCACTGTCCACTTC
mCCL5 FWD	GCTGCTTTGCCTACCTCTCC
mCCL5 REV	TCGAGTGACAAACACGACTGC
mCCL2 FWD	CCTGCTGCTACTCATTCACCAG
mCCL2 REV	AAACTACAGCTTCTTTGGGACACC
mKC FWD	CTGGGATTCACCTCAAGAACATC
mKC REV	CAGGGTCAAGGCAAGCCTC
mActb FWD	CCTTCTTGGGTATGGAATCCTGTG
mActb REV	CAGCACTGTGTTGGCATAGAGG
mItln1 FWD	TCTCTATTTCCTGCGCACGAA
mItln1 REV	GCTGACTGGACCAGCGATCAC
mRetnlb FWD	AGCTCTCAGTCGTCAAGAGCCTAA
mRetnlb REV	CACAAGCACATCCAGTGACAACCA
mAng4 FWD	TCTCCAGGAGCACACAGCTA
mAng4 REV	AAGGACATGGGCTCATTGTC
mPLA2g2a FWD	CTGTTGCTACAAGAGCCTGG
mPLA2g2a REV	TTTTCTTGTTCCGGGCGAAA
mS100A8 FWD	TCAAGACATCGTTTGAAAGGAAATC
mS100A8 REV	GGTAGACATCAATGAGGTTGCTC
univ16SrRNA FWD	GTGGTGCATGGTTGTCGTCA
univ16SrRNA REV	ACGTCGTCCCCACCTTCCTC
